# High-resolution structure of the alcohol dehydrogenase domain of the bifunctional bacterial enzyme AdhE

**DOI:** 10.1107/S2053230X20010237

**Published:** 2020-08-19

**Authors:** Liyana Azmi, Eilis C. Bragginton, Ian T. Cadby, Olwyn Byron, Andrew J. Roe, Andrew L. Lovering, Mads Gabrielsen

**Affiliations:** a Institute of Infection, Immunity and Inflammation, University of Glasgow, University Avenue, Glasgow G12 8QQ, United Kingdom; b School of Biosciences, University of Birmingham, Edgbaston, Birmingham B15 2TT, United Kingdom; c CRUK Beatson Institute, Switchback Road, Glasgow G61 1BD, United Kingdom

**Keywords:** alcohol dehydrogenase, AdhE, *Escherichia coli*

## Abstract

The bifunctional alcohol/aldehyde dehydrogenase (AdhE) comprises both an N-terminal aldehyde dehydrogenase (AldDH) and a C-terminal alcohol dehydrogenase (ADH). *In vivo*, full-length AdhE oligomerizes into long oligomers known as spirosomes. However, structural analysis of AdhE is challenging owing to the heterogeneity of the spirosomes. Therefore, the domains of AdhE are best characterized separately. Here, the structure of ADH from the pathogenic *Escherichia coli* O157:H7 was determined to 1.65 Å resolution. The dimeric crystal structure was confirmed in solution by small-angle X-ray scattering.

## Abstract 2

The crystal structure of the alcohol dehydrogenase domain of AdhE from *Escherichia coli* is reported.

## 1. Introduction   

The 96 kDa bifunctional alcohol/aldehyde dehydrogenase (AdhE) is an intriguing protein by virtue of its structure and function. Functionally, AdhE is involved in a number of metabolic processes: mixed-acid fermentation, glycolysis, metabolism of l-threonine (Létoffé *et al.*, 2017 ▸), purine and pyrimidine (Müller *et al.*, 2012 ▸), and degradation of ethanol­amine (Kofoid *et al.*, 1999 ▸). AdhE is a crucial enzyme primarily in alcohol metabolism and catalyzes the conversion of the high-energy substrate acetyl-CoA to acetaldehyde and subsequently to ethanol. Structurally, full-length AdhE comprises an N-terminal aldehyde dehydrogenase (AldDH) and a C-terminal alcohol dehydrogenase (ADH) domain, and self-assembles *in vivo* into spiral-like structures known as spirosomes (Kawata *et al.*, 1976 ▸; Laurenceau *et al.*, 2015 ▸; Ueki *et al.*, 1982 ▸). The helical structure of spirosomes is speculated to enhance the enzymatic activity of AdhE as well as to protect the protein from its toxic intermediate product, acetaldehyde (Extance *et al.*, 2013 ▸; Kim *et al.*, 2019 ▸). Ethanol generation by AdhE has been extensively studied in the context of biofuel production. Many fermentative and thermophilic bacteria such as *Thermoanaerobacterium saccharo­lyticum*, *Clostridium thermocellum* (Lo *et al.*, 2015 ▸) and *Geobacillus thermogluco­sidasius* (Hills, 2015 ▸) have been used as model organisms in which AdhE was genetically modified to improve the ethanol yield. Point mutations in AdhE introducing different cofactor specificities have been found to regulate ethanol generation (Tian *et al.*, 2019 ▸; Zheng *et al.*, 2015 ▸). Based on work by Zheng *et al.* (2015 ▸), an Asp-to-Gly substitution in the ADH domain of *G. thermoglucosidasius* (residue 494 in *G. thermogluco­sidasius* numbering, corresponding to residue 487 in the *Escherichia coli* enzyme) was discovered to significantly increase ethanol production by changing the cofactor specificity from NADH to NADPH. The cofactor-binding region of AdhE is located within the ADH domain, suggesting the importance of the ADH domain with regard to improving ethanol production. Solving the high-resolution structure of the ADH domain could provide important insights into how this mutation leads to the observed differential cofactor occupancies. A structure could also provide clues about the basis of spirosome assembly. Here, we describe the atomic structure of the cofactor-bound ADH domain from *E. coli* determined by X-ray crystallo­graphy to 1.65 Å resolution. The structure of the apo form was also determined to 1.95 Å resolution. Solution data obtained via small-angle X-ray scattering agree with the crystal structures and confirm the dimeric structure of the ADH domain. Comparisons with other alcohol dehydrogenase structures revealed a loop that is involved in coordinating the domains of the bifunctional enzyme.

## 2. Materials and methods   

### 2.1. Macromolecule production   

The C-terminal part of the *adhE* gene from *E. coli* O157:H7 (encoding residues 451 to the C-terminus) was cloned into a p77 vector (p77-D2) which encodes a C-terminal His_6_ tag. The construct was transformed into *E. coli* BL21(DE3) cells, which were grown in lysogeny broth (LB) containing 100 mg ml^−1^ ampicillin at 37°C until an optical density (OD_600_) of 0.6–0.8 was reached, whereupon the cultures were induced with a 1 m*M* final concentration of isopropyl β-d-1-thiogalactopyranoside (IPTG) and left to grow at 28°C overnight. The cells were harvested and resuspended in 20 m*M* Tris pH 7.5, 500 m*M* NaCl, 5%(*v*/*v*) glycerol (buffer *A*) with 20 m*M* imidazole.

The cells were sonicated in the presence of 10 mg DNAse (Sigma), 1 mg ml^−1^ EDTA-free protease inhibitors (Enzo Life Sciences) and 1 mg ml^−1^ lysozyme using 15 s on/off cycles, and the lysate was cleared by centrifugation and filtration. The cleared lysate was applied onto a 5 ml Ni^2+^ HisTrap column (GE Healthcare) that had been pre-equilibrated in buffer *A*, and was washed in buffer *A* plus 100 m*M* imidazole before the protein was eluted using an increasing gradient of imidazole. The purity of the protein was assessed by SDS–PAGE to be around 90–95%, and the protein-containing samples were dialyzed against buffer *A* with no imidazole but in the presence of TEV protease. Finally, the protein was loaded onto a Superdex 75 size-exclusion chromatography column (GE Healthcare) and concentrated using an Amicon Ultra 30 000 molecular-weight cutoff centrifugal filter (Millipore) to 10 mg ml^−1^. The final yield of the protein was 30 mg of purified protein from 1 l of culture. Macromolecule-production information is summarized in Table 1 ▸.

### 2.2. Crystallization   

Initial crystallization screens of purified ADH samples in buffer *A* at 10 mg ml^−1^ (based on the absorbance at 280 nm using an extinction coefficient of 0.838 *M* ^−1^ cm^−1^) were set up against the commercial ProPlex screen (Molecular Dimensions) using the sitting-drop vapour-diffusion technique. Rod-shaped crystals grew in 0.15 *M* ammonium sulfate, 0.1 *M* MES pH 6, 15%(*w*/*v*) PEG 4000 (condition 1-22 from ProPlex). To capture crystals containing the cofactor NAD, 0.5 m*M* NAD was added to the protein before the mixture was set up against the ProPlex screen. Rod-shaped crystals initially grew in 0.2 *M* lithium sulfate, 0.1 *M* MES pH 6.0, 20%(*w*/*v*) PEG 4000 (condition 1-28 from ProPlex), which was then optimized to 0.2 *M* lithium sulfate, 0.1 *M* MES pH 5.75, 14%(*w*/*v*) PEG 4000. A small fraction of the crystal was broken off for subsequent data collection. Both crystal forms grew within 48 h. Crystallization information is summarized in Table 2 ▸.

### 2.3. Data collection and processing   

Crystals were plunge-frozen in liquid nitrogen using 40%(*v*/*v*) ethylene glycol as a cryoprotecting agent. The *P*4_3_2_1_2_1_ data were collected on beamline I04-1 and the *P*2_1_2_1_2_1_ data were collected on beamline I03 at Diamond Light Source (DLS), Didcot, UK using a PILATUS 6M detector (Dectris, Switzerland). Data were collected with 0.2° oscillations for a total of 1200 images at wavelengths of 0.91741 Å and 0.97957 Å for the NAD-bound and apo crystals, respectively. Data were processed using *MOSFLM* and scaled and merged using *SCALA* from *CCP*4 (Winn *et al.*, 2011 ▸). Data-collection and processing statistics are summarized in Table 3 ▸.

### 2.4. Structure solution and refinement   

Molecular replacement used the structure of ADH from *G. thermoglucosidasius* (PDB entry 3zdr; Extance *et al.*, 2013 ▸) as a model in *Phaser* (McCoy *et al.*, 2007 ▸). Refinement was carried out in *Phenix* (Liebschner *et al.*, 2019 ▸), with visual inspection and manipulations in *Coot* (Emsley *et al.*, 2010 ▸). Structural superpositions were performed using *LSQMAN* (Kleywegt & Jones, 1994 ▸). Refinement statistics are summarized in Table 4 ▸.

### 2.5. Small-angle X-ray scattering (SAXS) data collection   

SAXS data were collected on beamline B21 at DLS with a camera length of 4.01 m at 12.4 keV using a PILATUS 2M detector (Dectris, Switzerland) at a wavelength of 0.1 nm. 50 µl ADH at a concentration of 10 mg ml^−1^ in buffer *A* was loaded onto a Shodex KW-403 (molecular-mass separation range 10–700 kDa) size-exclusion chromatography column (Showa Denko, Japan) at 0.16 ml min^−1^ using an Agilent 1200 HPLC system. 131 successive 1.0 s frames of SAXS data were recorded. The data were analysed using *ScÅtter* (http://www.bioisis.net) as follows. The estimated radius of gyration (*R*_g_) was plotted along with the integral of the ratio of the signal to background. A region showing a low signal-to-background ratio (low estimated *R*_g_) was picked and selected as buffer, and subtracted from regions showing higher constant *R*_g_ values (indicating monodispersity) and treated as samples. Successive SAXS analysis was performed using *ATSAS* 2.8 (Franke *et al.*, 2017 ▸). *R*_g_ was determined using the Guinier approximation (Guinier, 1939 ▸). The pairwise distance distribution function *p*(*r*) was determined using an indirect Fourier transformation method in *GNOM* (Svergun, 1992 ▸). Iterative estimation of *p*(*r*) allows an alternative estimation of *R*_g_ and the maximum particle dimension *D*_max_. Rigid-body modelling of both the SAXS curve and the crystal structure of ADH were assessed using the *Fast SAXS Profile Computation with Debye Formula* (*FoXS*) server (https://modbase.compbio.ucsf.edu/foxs/; Schneidman-Duhovny *et al.*, 2010 ▸). All of the SAXS data analysed for ADH were deposited in SASBDB (Valentini *et al.*, 2015 ▸) as entry SASDC72.

## 3. Results and discussion   

**Figure 1:**
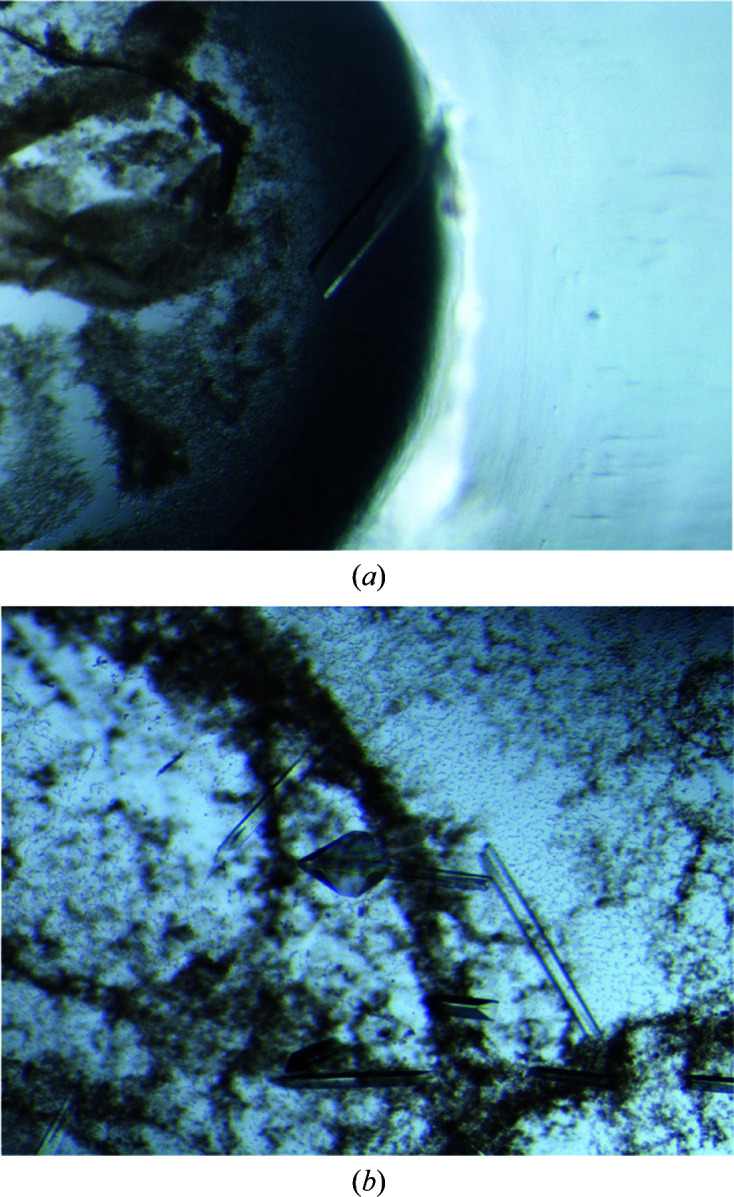
Crystals of ADH from *E. coli*. (*a*) Orthorhombic space group *P*2_1_2_1_2_1_, (*b*) tetragonal space group *P*4_3_2_1_2_1_.

The alcohol dehydrogenase domain of AdhE (ADH) from *E. coli* O157:H7 was crystallized in the apo form and bound to the cofactor NAD in two different crystal forms: the orthorhombic space group *P*2_1_2_1_2_1_ with unit-cell parameters *a* = 71.03, *b* = 96.73, *c* = 122.89 Å and the tetragonal space group *P*4_3_2_1_2 with unit-cell parameters *a* = *b* = 97.11, *c* = 233.36 Å, respectively (Fig. 1 ▸). The data were processed to 1.95 and 1.65 Å resolution, respectively, based on the relevant statistics (Table 3 ▸) and the structures were solved via molecular replacement. The electron density covers the majority of the residues from residue 450 (using full-length *E. coli* AdhE numbering) to residue 869. There is a gap in the electron density for both structures from residues 755 to 769, as well as missing electron density for the last 20 residues. It is assumed that these regions are particularly flexible or disordered.

The two forms superimpose well, with a root-mean-square deviation (r.m.s.d.) of 0.32 Å over 404 Cα atoms, and interestingly there are no obvious local differences in the binding site for NAD. The following discussion will therefore focus on the NAD-bound structure unless otherwise stated. The structure of ADH is similar to that of the homologous domain from *G. thermoglucosidasius* AdhE (gADH; Extance *et al.*, 2013 ▸; PDB entry 3zdr), with an r.m.s.d. of 1.2 Å over 373 Cα atoms when superposed. The main differences between ADH from *E. coli* and the previous structure are around the disordered loop, which is also observed in gADH, the region between residues 578 and 587, and an inserted proline at position 787 in the *E. coli* structure compared with the structure of gADH. Otherwise the folds are highly conserved.

**Figure 2:**
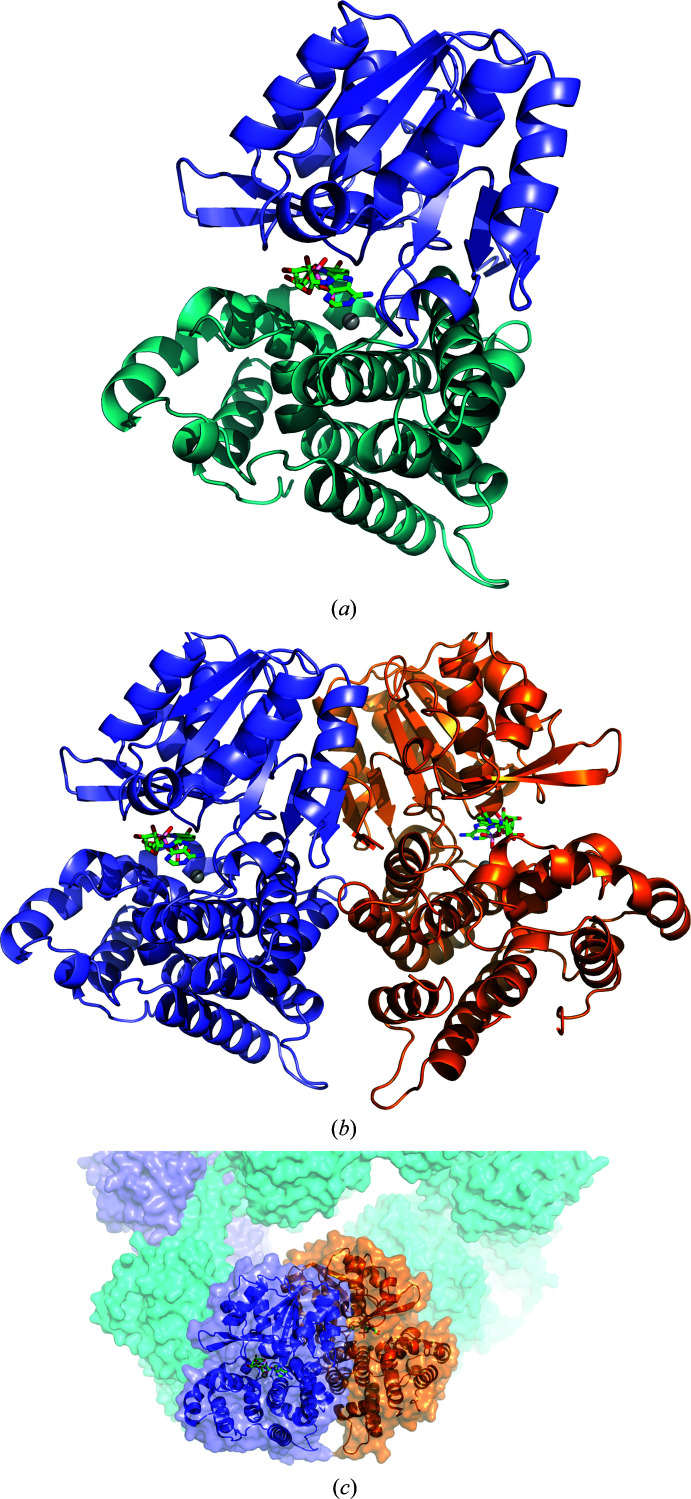
(*a*) Cartoon representation of a monomer of the ADH domain of AdhE from *E. coli*. The two subdomains are coloured slate (N-terminal) and teal (C-terminal), with NAD represented by green sticks and Fe^2+^ ions by grey spheres. (*b*) Cartoon representation of the oligomeric assembly, in which the two subunits forming the dimer are coloured separately. (*c*) Crystal structure of ADH superposed on the full-length spirosome of AdhE (PDB entry 6ahc; Kim *et al.*, 2019 ▸).

Structurally, ADH comprises two subdomains: an N-terminal Rossmann-like fold (residues 450–640), in which two parallel β-sheets are sandwiched between five α-helices, and a bundle of 11 α-helices (Fig. 2 ▸ *a*). The surface between the two subdomains consists of a tight network of hydrogen bonds, as well as a salt bridge between Arg463 and Glu701. The interface area is made up of 1300 Å^2^, corresponding to around 13% of the overall surface-accessible surface area of the ADH subunit.

**Figure 3:**
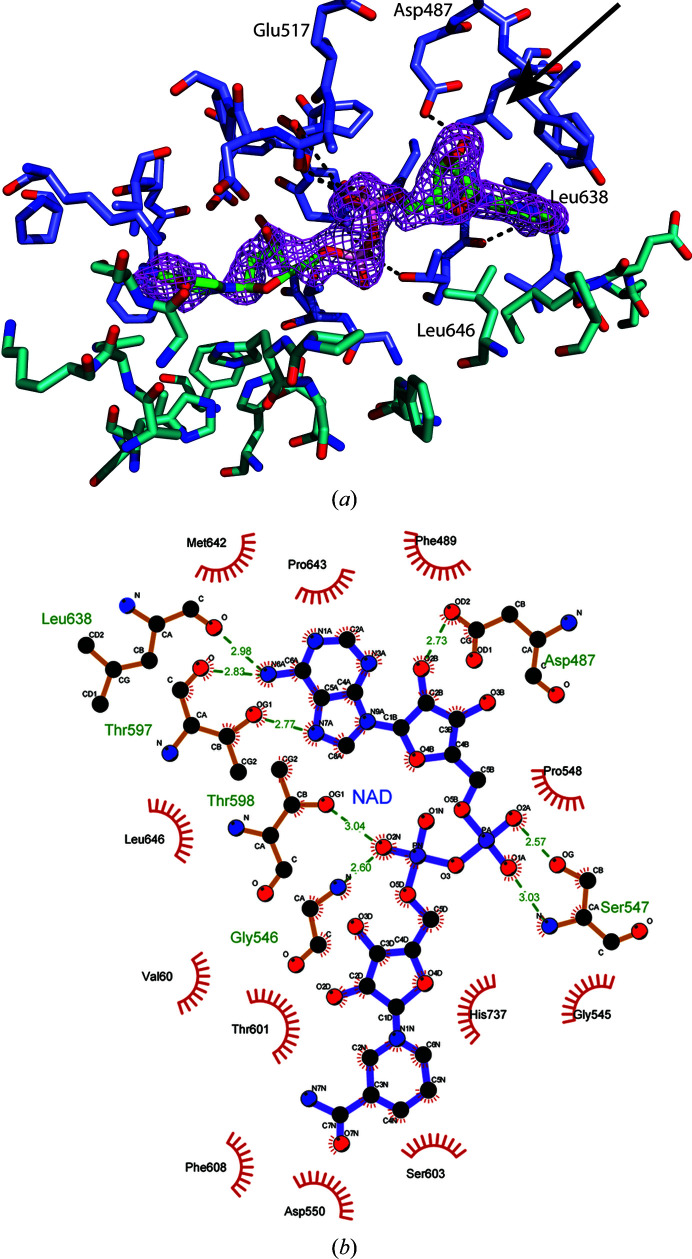
Close-up of the pocket where NAD is bound to the ADH structure, with a similar colour scheme as in Fig. 1 ▸(*a*). (*a*) Calculated polder maps at 3σ, showing the electron density for the ligand at an occupancy of 0.6; the arrow highlights where a clash would occur if NAD were substituted by NADP. (*b*) *LIGPLOT* figure of NAD and interactions with the protein environment.

The electron-density maps of ADH crystallized in the tetragonal space group showed extraneous features in the region of the conserved NAD-binding site, which is situated between the two subdomains of ADH (Fig. 2 ▸ *a*). Consequently, NAD was modelled into these features and refined to an occupancy of 0.6. Polder maps were calculated (Liebschner *et al.*, 2017 ▸) to confirm the positioning of the ligand (Fig. 3 ▸ *a*). NAD sits in the cleft formed between the two subdomains comprising ADH and the binding is mostly hydrophobic, with hydrogen bonds formed between Asp487, Gly546, Thr597 and Leu638 and the adenosine part of the NAD moiety and with Ser547 coordinating the phosphates (Fig. 3 ▸ *b*). The benzamide part of the NAD molecule is less ordered in the electron density, and it is likely that this part does not form strong interactions with ADH, allowing the moiety to exhibit a number of conformations. This is reflected in the *B* factors of the NAD moiety, where the adenosine diphosphate has an average *B* factor of 26 Å^2^ and the ribose-benzamide end has an average *B* factor of 49 Å^2^, which is higher than the average *B* factor of the protein model of 28 Å^2^. NAD interactions correspond to 3% of the total accessible surface area of ADH. The conserved residue Asp487 has been demonstrated to be important for the preference for NAD over NADP in alcohol dehydrogenases (Zheng *et al.*, 2015 ▸). When looking at the structure (Fig. 3 ▸ *a*) it becomes apparent that the presence of the Asp side chain will cause a steric clash with the additional phosphate group present in NADP, whereas the previously reported Asp-to-Gly mutation (Zheng *et al.*, 2015 ▸) will allow the binding of both NAD and NADP.

**Figure 4:**
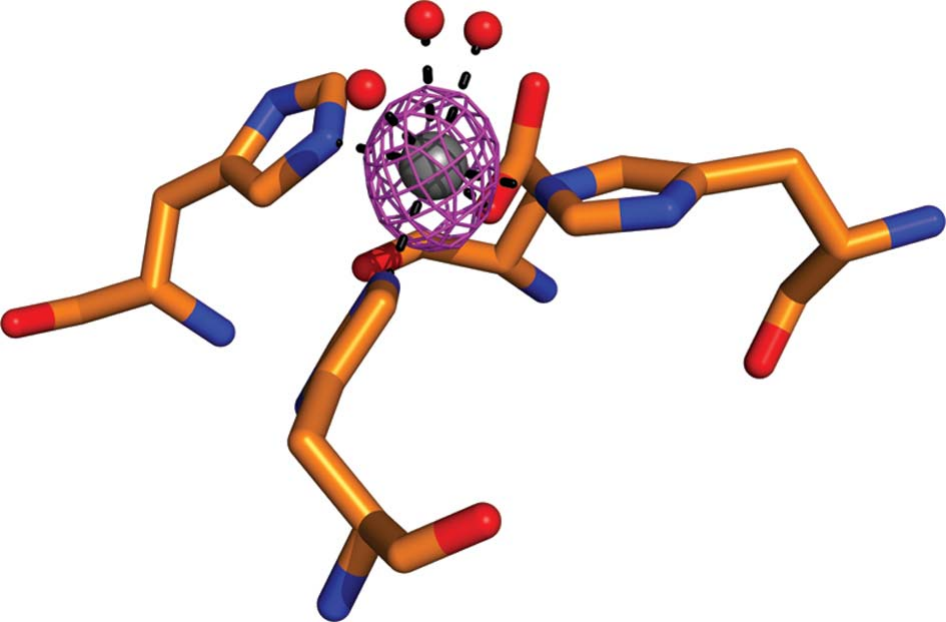
Fe^2+^ ion modelled in the electron density in the conserved metal ion site in the calculated polder map at 12σ.

Additional electron density was observed in the metal ion-binding site, as previously found in gADH, where it was identified as Zn^2+^. As ADH has been described as being reliant on binding to iron (Holland-Staley *et al.*, 2000 ▸) this density has been modelled as Fe^2+^, although it may also be a Zn^2+^ ion as observed in the homologue from *G. thermoglucosidasius*. The metal ion is coordinated by Asp653, His657, His723 and His737 with additional waters (Fig. 4 ▸).

### 3.1. Oligomeric state   

As has been shown previously, the alcohol dehydrogenase domain of AdhE forms homodimers that are essential for the formation of the larger full-length AdhE spirosomes. As in gADH, ADH crystallized as a dimer in the asymmetric unit, and the contacts between the two subunits comprise approximately 1550 Å^2^, which is 10% of the accessible surface area of each subunit. This is similar to the buried surface area in the interface between the two subdomains that make up an ADH monomer.

**Figure 5:**
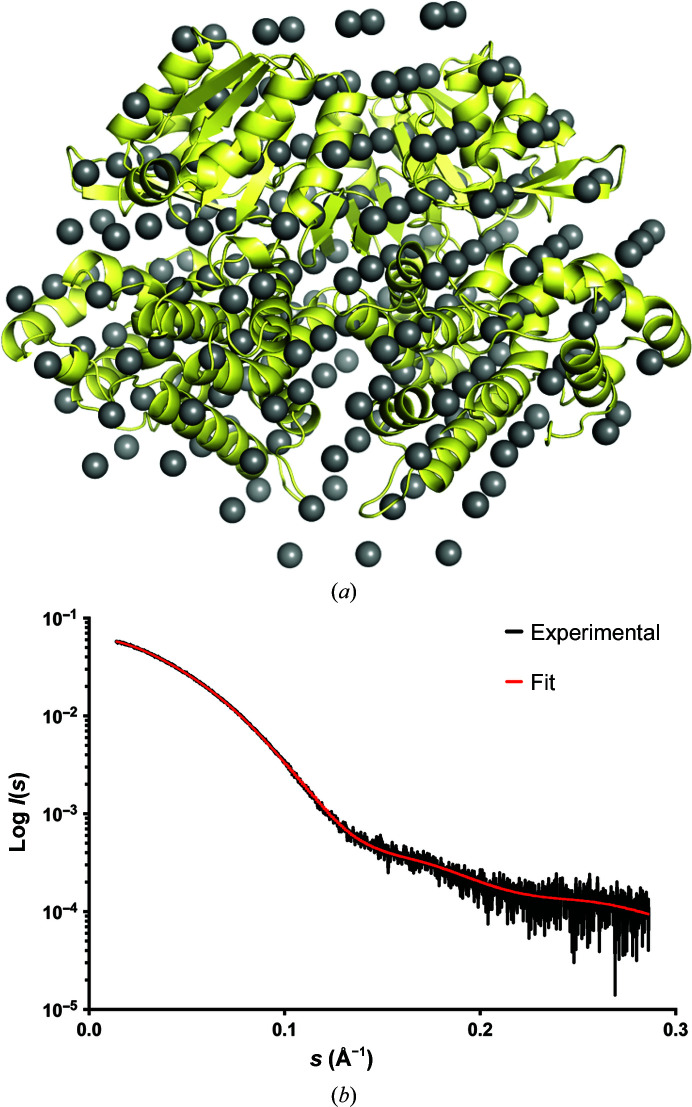
(*a*) Crystal structure of the apo form of ADH superposed on the *ab initio* surface envelope determined by SAXS. (*b*) Experimental data and fit of the *ab initio* surface envelope (SASDB ID SASDC72).

To confirm the dimerization of ADH, small-angle X-ray scattering experiments were undertaken. A linear Guinier region gave a radius of gyration (*R*_g_) of 32.5 Å, whereas the *R*_g_ calculated for the crystal structure is 28.1 Å. The discrepancy between the two values may be caused by the residues that are not accounted for by the electron density (the loop of residues 755–769 and the C-terminal residues 869–891). This difference in *R*_g_ values was not taken into consideration when calculating the *R*_g_ of the crystal structure, which could leave this value lower than it should be. Alternatively, these stretches of residues may be flexible or disordered in solution, which will add additional scattering and will be interpreted as a larger *R*_g_. The pairwise distance distribution function *p*(*r*) was calculated with *GNOM* (Svergun, 1992 ▸), using a *D*_max_ of 161 Å. *Ab initio* models were calculated using *DAMMIF* (Franke & Svergun, 2009 ▸). The crystal structure was superposed on the averaged and filtered model with a good fit (Fig. 5 ▸). Rigid-body fitting of the crystal structure against the experimental SAXS data using *FoXS* (Schneidman-Duhovny *et al.*, 2010 ▸) also gave a good fit, with a χ^2^ of 2.54, again demonstrating that the crystal structure is a good representation of ADH in solution. The *DAMMIF* model and SAXS data for ADH have been deposited in SASBDB (Valentini *et al.*, 2015 ▸) as entry SASDC72.

## 4. Discussion   

**Figure 6:**
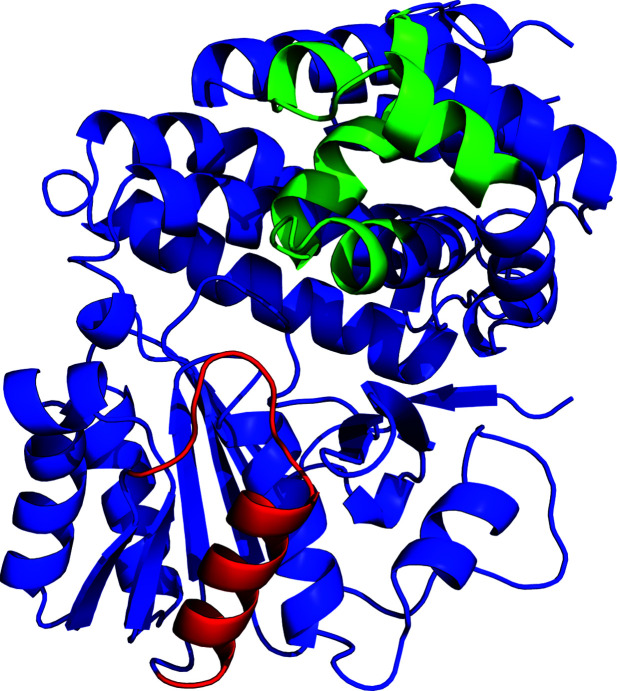
Structure of an ADH monomer, highlighting the regions that superpose less well when using *RAPIDO*. The N-terminal section with higher flexibility is in red and the C-terminal section with higher flexibility is in green.

Structures of the alcohol dehydrogenase domain from the bifunctional alcolhol/aldehyde dehydrogenase AdhE are reported here in the apo form and bound to the cofactor NAD at high resolutions. When searching for similar structures using the protein structure comparison service *PDBeFold* at EBI (Krissinel & Henrick, 2004 ▸), we found six structures (using a *Q*-value of 0.7 as a cutoff) determined by X-ray crystallography, which are all prokaryotic dehydrogenases with metal ions and NAD as cofactors (Table 5 ▸). When superposed over the Cα backbone, they all superpose with reasonable r.m.s.d. values of around 1.2 Å, suggesting that the subunit of ADH is structurally highly conserved, whereas the sequence identity between ADH and the individual dehydrogenases is between 30% and 35%. The structure that varies most is a lactaldehyde dehydrogenase from *E. coli* (PDB entry 1rrm; New York SGX Research Center for Structural Genomics, unpublished work), with an r.m.s.d. of 1.4 Å; the sequence identity between this protein and ADH is 31%. When using *RAPIDO*, a web server that superposes a number of protein structures (Mosca & Schneider, 2008 ▸) and identifies domains or part of domains that do not move versus those that do move, it is clear that the differences between the structures are located around a single helix in the N-terminal subdomain and a helix–turn–helix in the C-terminal domain (Fig. 6 ▸). It is clear that the structural conservation of this domain is high throughout.

Recently, a high-resolution structure of full-length AdhE was determined by the Song group (Kim *et al.*, 2019 ▸) using cryoEM, where they described that residue Phe670 (using *E. coli* K-12 numbering) is crucial for maintenance of the spirosome structure. Superposition of the ADH crystal structure with the ADH domain from the full-length AdhE structure gave an r.m.s.d. of 1.21 Å, indicating conservation of the dimeric ADH in the spirosomes. Residues 755–769 were unaccounted for in the ADH electron-density maps. However, in the full-length cryoEM structure they are present and interact with the AldDH domain of AdhE. These residues are also missing in the structure of gADH, but this loop does not exist in the other structures, which are all monofunctional. It appears that these residues are stabilized by the presence of AldDH and play a role in coordinating the two domains in relation to each other.

Kim *et al.* (2019 ▸) hypothesized that hydrophobic inter­actions surrounding Phe670 are crucial for the complete AdhE spirosome structure. Upon the substitution of Phe670 by Glu, the spirosome structure was disrupted (Kim *et al.*, 2019 ▸). Using *PISA* (Krissinel & Henrick, 2007 ▸) to calculate the interfaces involved in the formation of the ADH dimer, we observe that the number of atoms involved in the dimer interface is reduced by around 100 in the Phe670 mutant. Disruption of the hydrophobic interaction through the mutation of Phe670 to glutamic acid was found to break the spirosome assembly into mixtures of dimeric AdhE and other higher oligomeric spirosomes. The SAXS model of the mutant Glu670-AdhE (SASBDB ID SASDGN2; Kim *et al.*, 2019 ▸) shows the full-length AdhE dimers to be connected through the AldDH molecules rather than the ADH domains. This finding indicates the importance of the hydrophobic interactions of Phe670 in maintaining the helical structure of the spirosome and possibly the dimer conformation of ADH.

Finally, our data here present the high-resolution crystal structures of both apo and NAD-bound forms of the alcohol dehydrogenase domain of AdhE from *E. coli* O157:H7 and confirm the oligomeric state and solution structure using SAXS. We also show that the ADH fold is conserved even though there is low sequence identity, and that an inserted loop in the C-terminal part of ADH appears to be involved in coordination of the two domains of the bifunctional AdhE. With the availability of the crystal structure of ADH, future work could explore the mechanism of action of antivirulence compounds. Since AdhE has been shown to be important both as a tool for biofuel production as well as in virulence regulation (Beckham *et al.*, 2014 ▸), a complete mechanistic understanding would provide a better understanding of the mechanism of action of the protein and how it relates to both bacterial virulence and ethanol production.

## Supplementary Material

PDB reference: alcohol dehydrogenase domain of AdhE, 6sci

PDB reference: NAD-bound, 6scg

SASBDB reference: alcohol dehydrogenase domain of AdhE, SASDC72

## Figures and Tables

**Table 1:** Macromolecule-production information

Source organism	*E. coli*
DNA source	*E. coli*
Forward primer	ACCATGGACATGCTGTGGCATAAGCTGCC
Reverse primer	ACCATGGCGCGGATTTCTTC
Cloning vector	StrataClone PCR UA
Expression vector	p77
Expression host	*E. coli*
Complete amino-acid sequence of the construct produced	MDMLWHKLPKSIYFRRGSLPIALDEVITDGHKRALIVTDRFLFNNGYADQITSVLKAAGVETEVFFEVEADPTLSIVRKGAELANSFKPDVIIALGGGSPMDAAKIMWVMYEHPETHFEELALRFMDIRKRIYKFPKMGVKAKMIAVTTTSGTGSEVTPFAVVTDDATGQKYPLADYALTPDMAIVDANLVMDMPKSLCAFGGLDAVTHAMEAYVSVLASEFSDGQALQALKLLKEYLPASYHEGSKNPVARERVHSAATIAGIAFANAFLGVCHSMAHKLGSQFHIPHGLANALLICNVIRYNANDNPTKQTAFSQYDRPQARRRYAEIADHLGLSAPGDRTAAKIEKLLAWLETLKAELGIPKSIREAGVQEADFLANVDKLSEDAFDDQCTGANPRYPLISELKQILLDTYYGRDYVEGETAAKKEAAPAKAEKKAKKSAPWGAGGLEVLFQGPGAAHMHHHHHHHH

**Table 2:** Crystallization

	Apo	NAD-bound
Method	Sitting-drop vapour diffusion	Sitting-drop vapour diffusion
Plate type	CombiClover	CombiClover
Temperature (K)	289	289
Protein concentration (mg ml^−1^)	10	10
Buffer composition of protein solution	20 m*M* Tris pH 7.5, 500 m*M* NaCl, 5%(*v*/*v*) glycerol	20 m*M* Tris pH 7.5, 500 m*M* NaCl, 5%(*v*/*v*) glycerol, 0.5 m*M* NAD
Composition of reservoir solution	0.15 *M* ammonium sulfate, 0.1 *M* MES pH 6.0, 15%(*w*/*v*) PEG 4000	0.2 *M* lithium sulfate, 0.1 *M* MES pH 5.75, 14%(*w*/*v*) PEG 4000
Volume and ratio of drop	4 µl, 1:1 ratio	4 µl, 1:1 ratio
Volume of reservoir (µl)	140	140

**Table 3:** Data collection and processing Values in parentheses are for the outer shell.

	Apo	NAD-bound
Diffraction source	I03, DLS	I04-1, DLS
Wavelength (Å)	0.97957	0.91741
Temperature (K)	100	100
Detector	PILATUS 6M	PILATUS 6M
Crystal-to-detector distance (mm)	176	176
Rotation range per image (°)	0.2	0.2
Total rotation range (°)	240	240
Exposure time per image (s)	0.2	0.2
Space group	*P*2_1_2_1_2_1_	*P*4_3_2_1_2
*a*, *b*, *c* (Å)	71.03, 96.73, 122.89	97.14, 97.14, 233.43
α, β, γ (°)	90, 90, 90	90, 90, 90
*R*_meas_ (%)	8.5 (57.8)	10.5 (198.5)
*R*_p.i.m._ (%)	6.7 (45.3)	3.7 (77.7)
CC_1/2_	0.996 (0.718)	1.000 (0.577)
Mosaicity (°)	0.1	0.1
Resolution range (Å)	76.01–1.95	50.024–1.65
Total No. of reflections	59155	134582
No. of unique reflections	62248	134736
Completeness (%)	99.7	100
Multiplicity	4.4 (4.5)	17.7 (14.2)
〈*I*/σ(*I*)〉	10.1 (2.3)	19.1 (1.4)
Overall *B* factor from Wilson plot (Å^2^)	34.20	23.37

**Table 4:** Structure solution and refinement Values in parentheses are for the outer shell.

	Apo	NAD-bound
PDB code	6sci	6scg
Resolution range (Å)	76.01–1.95 (2.00–1.95)	50.02–1.65 (1.67–1.65)
Completeness (%)	99.7 (99.8)	99.9
σ Cutoff	*F* > 1.36σ	*F* > 1.36σ
No. of reflections, working set	59155 (4370)	127848 (4169)
No. of reflections, test set	3028 (210)	6731 (216)
Final *R*_cryst_	0.188 (0.257)	0.155 (0.260)
Final *R*_free_	0.207 (0.252)	0.181 (0.274)
Cruickshank DPI	0.132	0.09
No. of non-H atoms		
Protein	6264	12622
Ligand	2	1004
Water	109	812
R.m.s. deviations		
Bonds (Å)	0.011	0.013
Angles (°)	1.401	1.283
Average *B* factors (Å^2^)		
Protein	34.80	28.13
Ions	21.42	30.00
Ligand	NA	33.43
Water	27.80	40.36
Ramachandran plot		
Most favoured (%)	98.00	98.50
Allowed (%)	2.00	1.25
Outliers (%)	0	0.25

**Table 5:** PDB files used for structural alignment

PDB code	Protein	Species	R.m.s.d. (Å)	No. of C^α^ atoms	Reference
3bfj	1,3-Propanediol oxydoreductase	*Klebsiella pneumoniae*	1.2	348	Marcal *et al.* (2009 ▸)
3zdr	Alcohol dehydrogenase domain	*G. thermoglucosidasius*	1.2	373	Extance *et al.* (2013 ▸)
4fr2	Alcohol dehydrogenase	*Oenococcus oeni*	1.2	352	Elleuche *et al.* (2013 ▸)
3ox4	Alcohol dehydrogenase	*Zymomonas mobilis*	1.2	367	Moon *et al.* (2011 ▸)
2bl4	Lactaldehyde oxidoreductase	*E. coli*	1.3	356	Montella *et al.* (2005 ▸)
1rrm	Lactaldehyde oxidoreductase	*E. coli*	1.4	348	New York SGX Research Center for Structural Genomics (unpublished work)
